# Platelet Count as a Marker of Disease Severity in Pregnancy-Induced Hypertension: A Cross-Sectional Study

**DOI:** 10.7759/cureus.112576

**Published:** 2026-07-13

**Authors:** Aditi Dad, Pooja Saunakiya, Ayushi Tiwari, Maneesh Sulya, Sonal Gupta, Poorva Badkur

**Affiliations:** 1 Department of Pathology, Government Medical College, Ratlam, IND; 2 Department of Pathology, Gandhi Medical College, Bhopal, IND; 3 Department of Obstetrics and Gynaecology, Gandhi Medical College, Bhopal, IND

**Keywords:** mean arterial pressure, platelet count, preeclampsia, pregnancy-induced hypertension, thrombocytopenia

## Abstract

Background and objective

Pregnancy-induced hypertension (PIH) is a major contributor to maternal and perinatal morbidity and mortality. Platelet count has been proposed as a simple hematological marker of disease severity. This study aimed to evaluate platelet count as a marker of PIH severity and examine its correlation with mean arterial pressure (MAP).

Methods

This hospital-based cross-sectional analytical study included 220 pregnant women at more than 20 weeks of gestation: 110 normotensive controls and 110 PIH cases, comprising 48 with gestational hypertension (GHTN), 39 with preeclampsia (PE), and 23 with severe preeclampsia (SPE). Continuous variables were summarized as the median (interquartile range (IQR)) because of their non-normal distribution. Platelet counts were measured using an automated hematology analyzer. Blood pressure and MAP were recorded using standard methods. Group comparisons were performed using appropriate non-parametric tests. The association between platelet count and MAP was also assessed. Exploratory receiver operating characteristic (ROC) analysis was performed to assess the discriminatory performance of the platelet count.

Results

Median age of the participants was similar across groups (p = 0.956). Median gestational age was significantly lower in women with PE and SPE than in normotensive women (p = 0.002). Platelet count showed a stepwise decline across groups, from 3.3 (2.9-3.6) ×10⁵/µL in normotensive women to 2.2 (2.1-2.4) ×10⁵/µL in GHTN (1.7 (1.4-1.9) ×10⁵/µL in PE, and 1.2 (1.1-1.3) ×10⁵/µL in SPE; p < 0.0001; η² ≈ 0.70). MAP increased progressively with disease severity and showed a strong inverse correlation with platelet count (Spearman's ρ = −0.774, p < 0.001). Exploratory ROC analysis showed good discriminatory performance of platelet count for PIH versus normotensive pregnancy (area under the curve (AUC): 0.95; optimal cut-off: 2.5×10⁵/µL; sensitivity: 90.0%; specificity: 90.9%) and for SPE versus non-severe disease (AUC: 0.96; optimal cut-off: 1.7×10⁵/µL; sensitivity: 95.7%; specificity: 86.8%).

Conclusions

Platelet count decreased progressively with increasing severity of PIH and was strongly inversely associated with MAP. Exploratory ROC findings suggest that platelet count has potential diagnostic utility, but the results should be interpreted cautiously because of the cross-sectional, single-center design. Prospective multicenter validation is needed before platelet count can be recommended as a standalone clinical marker.

## Introduction

Pregnancy-induced hypertension (PIH), encompassing gestational hypertension (GHTN), preeclampsia (PE), and eclampsia, is a leading obstetric complication worldwide and accounts for approximately 14-16% of maternal deaths [1,2]. India faces a high PIH burden, with PE reported in approximately 8-10% of pregnancies. PIH, particularly PE, results from abnormal placentation with inadequate spiral artery remodeling, leading to uteroplacental ischemia [3]. Placental hypoxia promotes the release of anti-angiogenic factors, including soluble fms-like tyrosine kinase-1 and endoglin, thereby causing widespread maternal endothelial dysfunction. This is characterized by reduced nitric oxide-mediated vasodilation, increased vascular permeability, inflammation, and a prothrombotic state due to platelet and coagulation activation. Clinically, these changes manifest as hypertension, proteinuria, and multiorgan involvement. Severe disease may progress to eclampsia, HELLP (Hemolysis, Elevated Liver enzymes and Low Platelets) syndrome, or disseminated intravascular coagulation due to consumptive coagulopathy [4].

Given the central role of coagulopathy in PIH, platelet count and derived platelet indices have emerged as important markers of disease progression [5]. Thrombocytopenia, the most common hematological abnormality in PE, reflects increased platelet activation and consumption secondary to endothelial injury and microthrombus formation. As disease severity increases, platelet counts decline, while mean platelet volume (MPV) and platelet distribution width (PDW) rise due to the release of larger, immature platelets from the bone marrow. Several studies have demonstrated significantly lower platelet counts in preeclamptic women compared with normotensive controls, with these alterations correlating with blood pressure and disease severity [5,6].

Tracking these hematological changes may help clinicians assess the progression of PIH and promptly identify complications such as HELLP syndrome or disseminated intravascular coagulation. Hence, this study aimed to evaluate the association between platelet count and the severity of PIH by comparing platelet counts across normotensive, GHTN, PE, and severe preeclampsia (SPE) groups. The secondary objective was to determine the correlation between platelet count and mean arterial pressure (MAP).

## Materials and methods

This was a hospital-based cross-sectional analytical study conducted in the Departments of Pathology and Obstetrics and Gynecology at a tertiary care teaching hospital in central India between June 2023 and October 2024, after obtaining approval from the Institutional Ethics Committee vide letter number 18926/MC/IEC/2023.

A total of 220 participants were included, comprising 110 normotensive pregnant women and 110 PIH cases. The PIH group included 48 cases of GHTN, 39 cases of PE, and 23 cases of SPE. Consecutive sampling of eligible antenatal and inpatient women after 20 weeks of gestation was performed. Pregnant women attending antenatal clinics or admitted to obstetric wards after 20 weeks of gestation were screened for eligibility and enrolled after obtaining informed written consent. Participants were categorized into a normotensive group and a PIH group comprising GHTN, PE, and SPE.

PIH was defined as new-onset hypertension occurring after 20 weeks of gestation in a previously normotensive woman, with systolic blood pressure (SBP) ≥ 140 mmHg and/or diastolic blood pressure (DBP) ≥ 90 mmHg. Severity of PE was classified according to the American College of Obstetricians and Gynecologists (ACOG) Practice Bulletin criteria. Women with conditions known to affect platelet counts, including pre-existing chronic hypertension, hematological or coagulation disorders, liver or renal disease, diabetes mellitus, autoimmune disorders, multiple gestation, antepartum hemorrhage, intrauterine fetal demise, or use of anticoagulant or antiplatelet therapy, were excluded.

SPE was classified using standard clinical and laboratory criteria independent of platelet count. Platelet count was measured only as an outcome variable and was not used for group allocation or severity classification, thereby avoiding circular reasoning. Blood pressure was measured using a calibrated sphygmomanometer, and proteinuria was assessed by urine albumin estimation. Blood pressure was recorded by trained staff after the participant had rested in the sitting position for at least five minutes, using an appropriately sized cuff on the upper arm. Two readings were obtained four to five minutes apart, and the average of the two values was recorded for analysis. MAP was calculated using the following formula:

\[ \mathrm{MAP} = \frac{\mathrm{SBP} + 2\mathrm{DBP}}{3} \]

Under strict aseptic precautions, venous blood samples were collected for hematological assessment. Approximately 3-4 mL of blood was drawn into EDTA vacutainers for complete blood count. All samples were processed within recommended time limits to minimize pre-analytical variability. Platelet count was measured using an automated cell counter (Mindray 5-part analyzer; exact model number: BC-5150; Mindray, Shenzhen, China). Peripheral blood smears were examined to evaluate platelet morphology and exclude pseudothrombocytopenia. All investigations were conducted in the institutional laboratory in accordance with standard operating procedures and internal quality control protocols.

Proteinuria was assessed by urine dipstick testing. A reading of ≥ 1+ was considered significant proteinuria. Where available, the dipstick result was confirmed using repeat testing or laboratory confirmation as per institutional practice. Internal quality control was performed daily using commercial control materials at low, normal, and high levels, and results were accepted only when control values fell within the manufacturer’s specified range. External quality assurance/proficiency testing was followed according to institutional laboratory policy. Blood samples were collected within 24 hours of diagnosis/admission and, where feasible, before initiation of magnesium sulfate or antihypertensive therapy. Clotted or otherwise abnormal samples were repeated whenever possible, and unresolved specimens were excluded from analysis.

Data were entered into Microsoft Excel and analyzed using SPSS version 20.0 (IBM Corp., Armonk, NY). Continuous variables were summarized as median (interquartile range (IQR)) and compared using the Kruskal-Wallis test followed by Dunn’s post-hoc test, as appropriate. Correlation between platelet count and MAP was assessed using Spearman’s rank correlation test. A p-value < 0.05 was considered statistically significant.

## Results

A total of 220 pregnant women were analyzed, comprising 110 normotensive controls, 48 women with GHTN, 39 with PE, and 23 with SPE, exactly matching the original group sizes. Median maternal age was similar across groups: 25 years (IQR: 22-28) in normotensive women, 25 (23-27.5) in GHTN, 24 (23-27) in PE, and 25 (22.5-29) in SPE; the Kruskal-Wallis test showed no significant difference (H = 0.32, p = 0.956, η² ≈ 0.0015). Median gestational age at assessment was highest in normotensive and GHTN women (39 weeks, IQR: 37-40 and 38-40, respectively) and lower in PE (38, IQR: 35.5-39) and SPE (38, IQR: 36-39), with a significant overall difference (H = 14.33, p = 0.0025, η² ≈ 0.065) (Table 1).

**Table 1:** Baseline characteristics of normotensive and PIH groups Kruskal–Wallis test with Dunn–Holm post-hoc comparisons; η² reported as effect size PIH: pregnancy-induced hypertension; IQR: interquartile range; GHTN: gestational hypertension; PE: preeclampsia; SPE: severe preeclampsia

Group	N	Age, years, median (IQR)	Gestational age, weeks, median (IQR)
Normotensive	110	25 (22–28)	39 (37–40)
GHTN	48	25 (23–27.5)	39 (38–40)
PE	39	24 (23–27)	38 (35.5–39)
SPE	23	25 (22.5–29)	38 (36–39)

Systolic and diastolic blood pressure increased progressively with the severity of PIH, and this pattern was reflected in MAP. Median MAP was 90.0 mmHg (IQR: 83.3-93.3) in normotensive women, 106.7 mmHg (IQR: 106.7-106.7) in GHTN, 113.3 mmHg (IQR: 106.7-116.7) in PE, and 120.0 mmHg (IQR: 116.7-125.0) in SPE, with a highly significant overall difference (Kruskal-Wallis H = 180.74, p ≈ 6.1 × 10⁻³⁹, η² ≈ 0.83). Bootstrap 95% confidence intervals (CIs) for the MAP median were 90.0-93.3 mmHg for normotensive women, 106.7-106.7 mmHg for GHTN, 110.0-116.7 mmHg for PE, and 116.7-123.3 mmHg for SPE (Table 2).

**Table 2:** MAP by group MAP was analyzed by the Kruskal–Wallis test with Dunn–Holm post-hoc comparisons; η² reported MAP: mean arterial pressure; IQR: interquartile range; CI: confidence interval; GHTN: gestational hypertension; PE: preeclampsia; SPE: severe preeclampsia

Group	N	MAP, mmHg, median (IQR)	95% CI for MAP median, mmHg
Normotensive	110	90.0 (83.3–93.3)	90.0–93.3
GHTN	48	106.7 (106.7–106.7)	106.7–106.7
PE	39	113.3 (106.7–116.7)	110.0–116.7
SPE	23	120.0 (116.7–125.0)	116.7–123.3

Platelet counts, expressed as ×10⁵/µL, showed the expected stepwise decline with increasing PIH severity. The median platelet count in normotensive women was 3.3 ×10⁵/µL (IQR: 2.90-3.60), decreasing to 2.2 ×10⁵/µL (IQR: 2.10-2.43) in GHTN, 1.7 ×10⁵/µL (IQR: 1.35-1.90) in PE, and 1.2 ×10⁵/µL (IQR: 1.10-1.30) in SPE. Kruskal-Wallis testing confirmed a highly significant overall difference (H = 152.53, p ≈ 7.5 × 10⁻³³), with a large effect size (η² ≈ 0.70), indicating that group membership explained a substantial proportion of the variability in platelet count. Bootstrap 95% confidence intervals for the median platelet count were 3.2-3.45 ×10⁵/µL for normotensive women, 2.1-2.30 ×10⁵/µL for GHTN, 1.4-1.80 ×10⁵/µL for PE, and 1.1-1.30 ×10⁵/µL for SPE, reinforcing the strong monotonic decline across severity categories (Table 3).

**Table 3:** Platelet count across normotensive and PIH severity groups Kruskal–Wallis test with Dunn–Holm post-hoc comparisons; η² reported PIH: pregnancy-induced hypertension; IQR: interquartile range; CI: confidence interval; GHTN: gestational hypertension; PE: preeclampsia; SPE: severe preeclampsia

Group	N	Platelet, ×10⁵/µL, median (IQR)	95% CI for median, ×10⁵/µL
Normotensive	110	3.3 (2.90–3.60)	3.2–3.45
GHTN	48	2.2 (2.10–2.43)	2.1–2.30
PE	39	1.7 (1.35–1.90)	1.4–1.80
SPE	23	1.2 (1.10–1.30)	1.1–1.30

**Figure 1:**
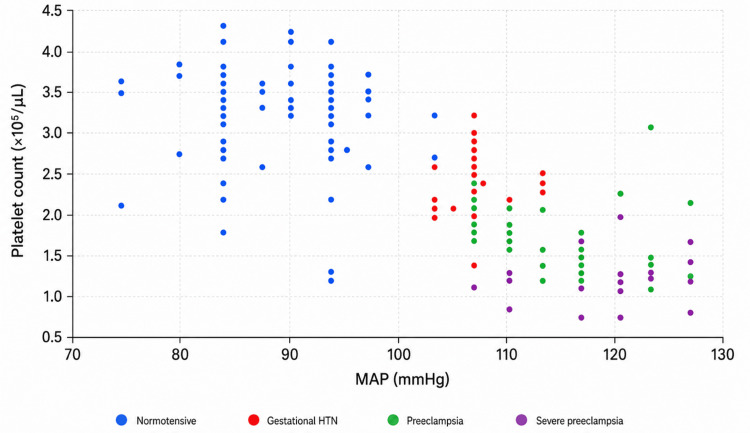
Scatter plot of platelet count vs. MAP MAP: mean arterial pressure; HTN: hypertension

Across the combined cohort, platelet count showed a strong inverse correlation with MAP (Spearman's ρ = −0.77, p ≈ 6.5 × 10⁻⁴⁵), consistent with more severe hemodynamic compromise being associated with lower platelet counts. This inverse relationship remained evident within PIH subgroups, although correlation coefficients were naturally attenuated by the narrower MAP range within each severity stratum (Figure 1).

Normotensive vs. PIH (GHTN + PE + SPE)

**Figure 2:**
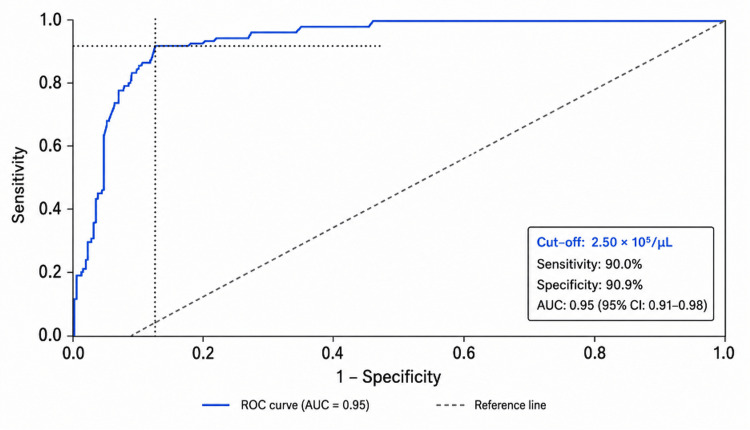
ROC curve for platelet count differentiating PIH from normotensive pregnancy ROC: receiver operating characteristic; PIH: pregnancy-induced hypertension; AUC: area under the curve; CI: confidence interval

When differentiating PIH cases from normotensive pregnancies, platelet count demonstrated excellent performance, with an area under the curve (AUC) of 0.95 (bootstrap 95% CI: 0.91-0.97). Using a cut-off of 2.5 ×10⁵/µL, with lower values indicating PIH, sensitivity was 90% and specificity was 91% for identifying PIH in this dataset. These findings suggest that modest reductions in platelet count may help identify women with hypertensive disorders of pregnancy, particularly in resource-limited settings where more advanced biomarkers may not be available; however, external validation is required (Figure 2).

SPE vs. non‑severe disease

**Figure 3:**
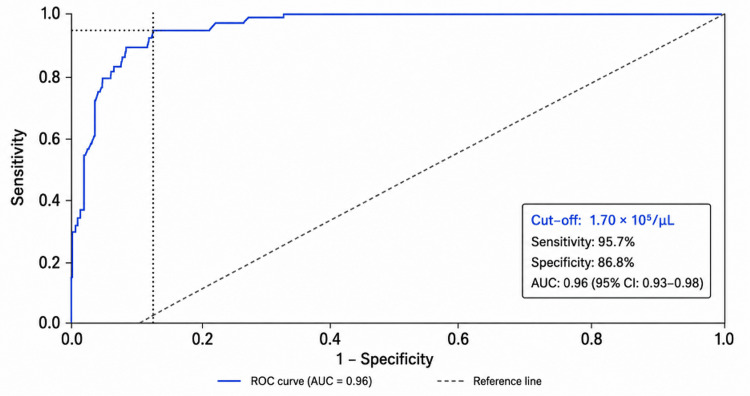
ROC curve for platelet count differentiating SPE ROC: receiver operating characteristic; SPE: severe preeclampsia; PIH: pregnancy-induced hypertension; AUC: area under the curve; CI: confidence interval

For differentiating SPE from all other groups (normotensive, GHTN, and PE), platelet count also performed very well, with an AUC of 0.96 (bootstrap 95% CI: 0.92-0.98). An optimal cut-off of 1.7 ×10⁵/µL yielded a sensitivity of 96% and specificity of 87% for identifying SPE. These high AUC values and favorable sensitivity/specificity profiles suggest that low platelet counts may have potential utility as part of a multimodal severity-assessment strategy; however, the receiver operating characteristic (ROC) findings are exploratory, drawn from a single-center cross-sectional cohort without outcome endpoints, and should therefore be interpreted cautiously (Figure 3).

## Discussion

The present cross-sectional study among 220 pregnant women comprised 110 normotensive, 48 GHTN, 39 mild PE, and 23 SPE cases. The mean maternal age ranged from 25.46 ± 3.76 to 26.78 ± 6.41 years (p = 0.956), suggesting that age was comparable across study groups. This is consistent with findings by Gupta et al. [7], who observed no significant difference in age between healthy pregnant women (mean = 5.26 ± 3.76 years) and those with PIH (mean = 26.87±4.53 years). Damani reported mean ages of 24.7 ± 5.6 years in PIH cases and 25.6 ± 5.8 years in controls (p > 0.05) [8]. However, they reported that a lower age at marriage (mean = 16.1 ± 3.5 years) was significantly associated with the incidence of PIH, suggesting that teenage pregnancy remains a significant risk factor in certain populations. Hailu et al. reported mean ages of 27.93 ± 5.80 years in PIH and 26.43 ± 4.99 years in normotensive women (p = 0.164) [9]. In contrast, Li et al. reported significantly higher maternal age in SPE (31.90 ± 6.35 years) compared to controls (29.74 ± 5.23 years; p < 0.001) [10].

A significant difference was observed in gestational age at delivery in the present study, with normotensive women delivering at a higher mean gestational age (38.43 ± 1.95 weeks) compared to those with mild PE (37.08 ± 2.64 weeks) and SPE (36.83±3.11 weeks) (p = 0.002). Li et al. similarly reported a significant reduction in gestational age from 38.71 ± 2.10 weeks in controls to 36.14 ± 2.90 weeks in mild PE and 34.02 ± 3.93 weeks in SPE (p<0.001) [10]. Gupta et al. [7] further supported this trend by reporting a higher incidence of preterm labor in PIH cases (32%) compared to controls (4%), while Sultana et al. [11] highlighted prematurity as a major contributor to adverse perinatal outcomes in PIH. In contrast, Reddy and Prasad [12] found comparable gestational ages between normal pregnancies (37 ± 2.5 weeks) and SPE (36.5 ± 2 weeks; p = 0.108). The earlier gestational age at delivery observed in PIH cases in our study may be explained by the need for timely obstetric intervention and planned early delivery to prevent maternal and fetal complications associated with increasing disease severity.

There was a significant decline in platelet count with increasing severity of hypertensive disorders of pregnancy in the present study. The mean platelet count showed a stepwise reduction from normotensive women (3.23 ± 0.58 ×10⁵/µL) to gestational hypertension (2.28 ± 0.36 ×10⁵/µL), mild PE (1.68 ± 0.43 ×10⁵/µL), and SPE (1.19 ± 0.32 ×10⁵/µL) (p < 0.0001). Post-hoc analysis further confirmed that each increase in disease severity was associated with a statistically significant reduction in platelet count. Similar findings were reported by Hailu et al. [9], who observed a significant reduction from 2.73 ± 0.34 ×10⁵/µL in controls to 2.01 ± 0.23 ×10⁵/µL in mild PIH and 1.35 ± 0.33 ×10⁵/µL in severe PIH, with significant intergroup differences. Gupta et al. [7] also demonstrated a progressive decline from 2.38 ×10⁵/µL in controls to 1.60 ×10⁵/µL in SPE and 0.99 ×10⁵/µL in eclampsia (p < 0.001).

Comparable trends were also reported by Reddy and Prasad [12], who noted decreasing platelet counts from 3.56 ×10⁵/µL in normal pregnancy to 1.81 ×10⁵/µL in SPE, and by Damani [8], who found significantly lower counts in PIH cases compared with controls (2.17 ×10⁵/µL vs 3.84 ×10⁵/µL; p = 0.003). Alemu et al. [13] reported significantly lower platelet counts in PE (1.50 ± 0.47 ×10⁵/µL) and eclampsia (1.34 ± 0.54 ×10⁵/µL) compared with controls (2.29 ± 0.39 ×10⁵/µL); however, they did not find a significant difference between PE and eclampsia (p = 0.130). In contrast, Sultana et al. [11] did not observe a consistent decline with severity, reporting normal platelet counts in 50% of GHTN and eclampsia cases. Li et al. [10] also reported no significant difference in platelet counts across groups (p = 0.194). These variations may be attributed to differences in study population, disease spectrum, and timing of laboratory assessment. Overall, our findings show that declining platelet count is associated with increasing severity of hypertensive disorders of pregnancy and may serve as a useful marker of disease progression.

The observed reduction in platelet counts with increasing disease severity in the present study highlights its potential utility as a simple and cost-effective prognostic marker. Similar observations were reported by Gupta et al. [7], who described platelet count as a simple and inexpensive prognostic tool directly related to PIH severity, and Reddy and Prasad [12], who highlighted its utility as an affordable screening test for early identification of PE. Hailu et al. [9], Alemu et al. [13], and other researchers [14,15] also demonstrated that the degree of thrombocytopenia correlates with disease severity, supporting its use in routine monitoring. However, Sultana et al. [11] noted that normal platelet counts may still be seen in severe disease, indicating that platelet count alone may not always predict severity. The proposed mechanisms as described in previous studies include increased platelet consumption due to endothelial injury, inflammatory activation, hypercoagulability, and increased thromboxane-mediated platelet aggregation [13,16].

We also observed a strong negative correlation between mean arterial pressure and platelet count (Spearman’s ρ = −0.774, p = 0.001), indicating that higher blood pressure levels were associated with lower platelet counts. Similar findings were reported by Hailu et al. [9], who demonstrated an even stronger negative correlation (r = −0.869, p < 0.001). Alemu et al. [13] also reported a significant inverse correlation, which strengthened with disease severity (ρ = −0.355, p = 0.005 in PE and ρ = −0.685, p < 0.001 in eclampsia). Reddy and Prasad [12] reported a weaker but significant negative correlation (r = −0.19, p = 0.003). Overall, our findings demonstrate that platelet count shows a significant inverse relationship with disease severity in hypertensive disorders of pregnancy and may serve as a useful marker for monitoring disease progression.

Our findings show a clear inverse relationship between platelet count and the severity of PIH, with progressive thrombocytopenia across gestational hypertension, PE, and SPE. This pattern is consistent with a previous study showing that platelet count decreases as disease severity increases, but the clinical interpretation should remain cautious because platelet count alone has limited standalone diagnostic value [17]. Our exploratory ROC analysis suggested that platelet count may have useful discriminatory ability for identifying PIH and severe PE in this cohort. However, the derived AUCs and cut-off values should be considered hypothesis-generating rather than definitive clinical thresholds. As the study was single-center and cross-sectional in design, prospective multicentre validation is required before platelet count can be recommended as an independent diagnostic or prognostic tool [18].

The main limitations of this study include its cross-sectional, single-center design, which precludes causal or prognostic inference, and the relatively small SPE subgroup, which may affect precision. In addition, not all potential confounders were adjusted for, and the ROC analysis was exploratory; therefore, the derived cut-off values require validation in larger prospective studies before clinical application. Although platelet count was not used to classify SPE, residual bias cannot be completely excluded.

## Conclusions

This study demonstrated a significant decline in platelet count with increasing severity of PIH, along with a negative correlation with MAP. Platelet count exhibited a clear inverse association with disease severity, showing progressively lower values across GHTN, PE, and SPE. Exploratory ROC analysis indicated that platelet count has the potential to discriminate between PIH and severe disease in this cohort; however, the identified cut-off values should be interpreted cautiously owing to the single-center, cross-sectional study design. Platelet count may therefore be considered an adjunctive marker of disease severity; however, large-scale prospective multicentre studies are required to validate its diagnostic and prognostic utility before routine clinical application.
